# Siglec-F is a novel intestinal M cell marker

**DOI:** 10.1016/j.bbrc.2016.08.055

**Published:** 2016-10-07

**Authors:** Nadezhda Gicheva, Matthew S. Macauley, Britni M. Arlian, James C. Paulson, Norihito Kawasaki

**Affiliations:** a Food and Health Institute Strategic Program, Institute of Food Research, Norwich, UK; b Department of Chemical Physiology, The Scripps Research Institute, La Jolla, CA, USA; c Department of Cell and Molecular Biology, The Scripps Research Institute, La Jolla, CA, USA; d Department of Immunology and Microbial Science, The Scripps Research Institute, La Jolla, CA, USA

**Keywords:** Siglec, M cell, Oral vaccine, Peyer's patch, M cell, microfold cell, PP, Peyer's patch, Siglec, sialic acid-binding immunoglobulin-like lectin, UEA-I, *Ulex europaeus* agglutinin-I

## Abstract

Intestinal microfold (M) cells are epithelial cells primarily present on Peyer's patches (PPs) in the small intestine. The ability of M cells to shuttle antigens into the PP for appropriate immune responses makes M cells a target for next-generation oral vaccine delivery. In this regard, discovery of M cell-specific receptors are of great interest, which could act as molecular tags for targeted delivery of cargo to M cells. Here, using a monoclonal antibody we generated to the Sialic acid-binding immunoglobulin-like lectin F (Siglec-F), we show that Siglec-F is expressed on mouse M cells in the small intestine. Immunohistochemical analysis of the PP tissue sections shows that Siglec-F is expressed on the surface of the M cell membrane exposed to the intestinal lumen. Anti-Siglec-F antibody injected into the mouse small intestine bound to M cells, demonstrating the potential to target M cells via Siglec-F.

## Highlights

• A new monoclonal antibody recognizing Siglec-F has been established.
• Siglec-F is expressed on mouse Peyer's patch microfold (M) cells.
• Siglec-F antibody binds to PP M cells *in vivo.*

## 1 Introduction

Intestinal microfold (M) cells are small intestinal epithelial cells localized on the Peyer's patches (PPs) [1]. M cells actively transport luminal antigens from foods, viruses, and bacteria into the PPs, leading to induction of the appropriate immune response [2]. Because of the role of M cells in antigen transport, targeted delivery of antigens to M cells has been recognized as an approach to enhance oral vaccine efficacy [3]. Towards this end, several well-defined cell surface markers on M cells have been investigated as targets to deliver antigens to M cells. Targets investigated to date include α1-2 fucosylated glycans expressed on murine M cells that are recognized by the monoclonal antibody NKM16-2-4 [4], and glycoprotein 2 (GP2), an M cell specific protein that can be targeted with an anti-GP2 [5], [6]. Oral administration of antigen conjugated to either the NKM16-2-4 or anti-GP2 antibody, greatly elevated serum IgG and intestinal IgA responses, providing a proof of concept for an M cell-targeted oral vaccine [4], [5].

Sialic acid-binding immunoglobulin-like lectins (Siglecs) are a family of proteins that recognize sialic acid residues on glycoproteins and glycolipids [7]. Siglecs were first characterized as immune cell receptors with functions including: regulation of cellular activation, tolerance induction, and pathogen recognition and uptake [7]. Recent studies, however, have revealed that several Siglecs are also expressed on non-immune cells, suggesting that Siglecs play roles beyond modulating immune cell responses. For example, Siglec-4 is well known to be expressed primarily on oligodendrocytes of the central nervous system [7], Siglec-6 in the placenta [8], Siglec-12 in the prostate and kidney [9], Siglec-5/14 on amniotic epithelium [10], and Siglec-5 on human M cells [11]. Due to their restricted expression pattern and efficient endocytic properties, Siglecs remain promising targets for delivering therapeutics to specific immune and non-immune cell types alike [12].

Siglec-F is a member of the Siglec family and was first identified as an eosinophil marker in mouse [7]. Siglec-F recognizes the NeuAcα2-3Gal[6S]β1-4GlcNAc motif printed on a synthetic glycan microarray [13], and it is proposed that binding of Siglec-F to a related glycan expressed on mouse lung potentiates eosinophil death in the lung during allergic asthma [14]. Accordingly, Siglec-F deficient mice exhibit increased numbers of lung-infiltrating eosinophils during an asthmatic state [15]. While these studies have established a suppressive function for Siglec-F on eosinophils, the role of Siglec-F in other cell-types, such as alveolar macrophages [16] and activated T cells [15], remains unclear.

We generated a monoclonal antibody recognizing Siglec-F to investigate its cell type expression and biological roles in mouse tissues and cells. Although attention to expression of Siglec-F has focused on immune cells in blood, spleen, and lymph nodes, we noted a single report of the presence of Siglec-F mRNA in mouse M cells [17]. As described here, we found that Siglec-F protein is indeed expressed on the luminal surface of small intestinal M cells. Furthermore, our Siglec-F antibody bound to M cells upon injection into the small intestine, demonstrating the potential of M cell targeting via Siglec-F.

## 2 Materials and methods

### 2.1 Animals

C57BL/6J WT and Siglec-F KO (Mutant Mouse Resource Research Center, University of California Davis, USA) mice and Lewis rats were maintained in the specific pathogen free animal facility at The Scripps Research Institute (La Jolla, USA). C57BL/6J and Balb/c mice were maintained in the specific pathogen free animal facility at the University of East Anglia (Norwich, UK). Animal use in this study was in the accordance with the guidelines of the Institutional Animal Care and Use Committee of The Scripps Research Institute and the UK Home Office.

### 2.2 Anti-Siglec-F monoclonal antibody generation

The anti-Siglec-F monoclonal antibody (rat IgG2b, clone 9C7) was generated as described previously [18]. Briefly, Lewis rats were immunized with Siglec-F-expressing CHO cells emulsified in complete Freund's adjuvant (Difco Laboratories). Following two boosts of the cells emulsified in incomplete Freund's adjuvant (Difco Laboratories), the immunized rats were sacrificed and the common iliac lymph nodes were harvested to generate hybridomas. The established hybridomas were screened for reactivity to Siglec-F-CHO cells and Siglec-F-expressing HEK293 cells by flow cytometry. The selected anti-Siglec-F expressing hybridoma was grown for 7–10 days, and the antibody was purified from the culture supernatant and labelled with Alexa Fluor-647 (Life Technologies).

### 2.3 Flow cytometry

PPs were harvested from mouse small intestine and vortexed in PBS vigorously. The supernatant was removed, and this washing step was repeated three times. Washed PPs were incubated with 10 ml of PBS containing 5 mM EDTA and 1 mM DTT for 30 min at 37 °C, in order to remove cells localized in the epithelia. The solution was passed through a 40 μm-cell strainer and centrifuged. The harvested cells were incubated with FcR-blocking antibody (Biolegend, clone 93) and stained with anti-M cell antibody NKM16-2-4 (MP Biomedicals) for 30 min at 4 °C, followed by Biotin-labelled anti-rat IgG2c (Southern Biotech). The cells were further stained with a cocktail of Alexa647-labelled anti-Siglec-F antibody, Alexa700-labelled anti-mouse CD45 (Biolegend, clone: 30-F11), PE-labelled anti-mouse CCR3 (Biolegend, J073E5), PE-Cy7-labelled CD11b (Biolegend, M1/70), FITC-labelled UEA-I (Vector lab), and Pacific blue-labelled streptavidin (Life Technologies). The stained cells were washed and resuspended in 0.3 μg/ml propidium iodide for dead cell exclusion prior to analysis on Fortessa or LSR II cell analyzers (BD Biosciences). Data were analyzed by FlowJo (Treestar).

### 2.4 Microscopy

Terminal ileum PPs were harvested from C57BL/6J mice, washed in 1x PBS, snap frozen in OCT (Bright CRYO-M-BED), and cryosectioned at 8-μm thickness. Sections were fixed for 5 min with −20 °C methanol, washed once with PBS containing 0.05% Tween-20 (PBS-T), and washed twice with PBS. All subsequent incubations were performed at RT. Sections were treated with 3% H_2_O_2_ in PBS for 30 min. After washing once with PBS-T and twice with PBS, slides were blocked with PBS containing 6% BSA and 10% normal mouse serum (Southern Biotech) for 30 min. Anti-mouse Siglec-F or isotype control (10 μg/ml) and UEA-I-FITC (20 μg/ml) were applied for 60 min. After washing, as described above, sections were incubated for 30 min with mouse anti-rat IgG2b-HRP (Southern Biotech, 2 μg/ml). Sections were washed once with PBS-T, washed four times with 1x PBS, and treated for 5 min with Cy-3 Plus TSA reagent (Perkin Elmer) according to the manufacturer's instructions. After washing once with PBS-T and four times with PBS, sections were incubated with anti-mouse M cell antibody NKM16-2-4 (25 μg/ml). After 60 min the slides were washed and treated with biotinylated anti-rat IgG2c (10 μg/ml) for 30 min, washed, and stained for 20 min with Alexa647 conjugated streptavidin and counterstained with DAPI (5 μg/ml). Sections were imaged with the Zeiss Axio Imager M2, and data were analyzed with ZEN 2 (blue edition).

### 2.5 Intestinal loop assay

Balb/c mice were anesthetized with a continuous flow of isoflurane. The small intestine was exposed via laparotomy, and an intestinal loop containing PPs was made using threads (Fig. 3A). Five micrograms of Alexa647-labelled anti-Siglec-F or isotype control antibody (Rat IgG2b, Biolegend) was injected into the loop. The mice were sacrificed 15 min after inoculation, and PPs were harvested. The Alexa647 signal associated with M cells was analyzed by flow cytometry.

## 3 Results and discussion

### 3.1 Generation of a Siglec-F monoclonal antibody

**Fig. 1:**
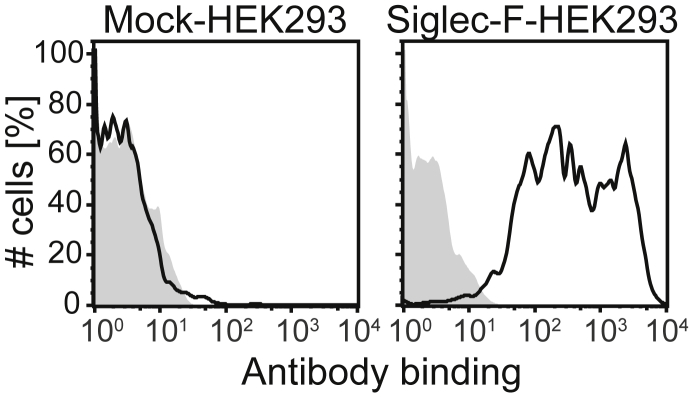
**A new anti-Siglec-F monoclonal antibody binds to Siglec-F expressing HEK293 cells**. HEK 293 cells expressing Siglec-F or mock transfectant were stained with Alexa 647-labelled Siglec-F (Black) or isotype control (Grey) antibody. The stained cells were analyzed by flow cytometry. Data are representative of two independent experiments with similar results.

In order to study Siglec-F expression profile on mouse tissues, we generated a monoclonal antibody to Siglec-F. Briefly, Lewis rats were immunized with Siglec-F expressing CHO cells. Hybridomas were established by fusing myeloma cell line with the lymphocytes from common iliac lymph nodes of the immunized animals. Hybridoma screening was performed based on the antibody reactivity to Siglec-F expressing CHO cells and HEK293 cells by flow cytometry. The established clone 9C7 produced a rat IgG2b monoclonal antibody, which binds to Siglec-F expressed on HEK293 cells (Fig. 1).

### 3.2 Expression of Siglec-F on PP M cells

**Fig. 2:**
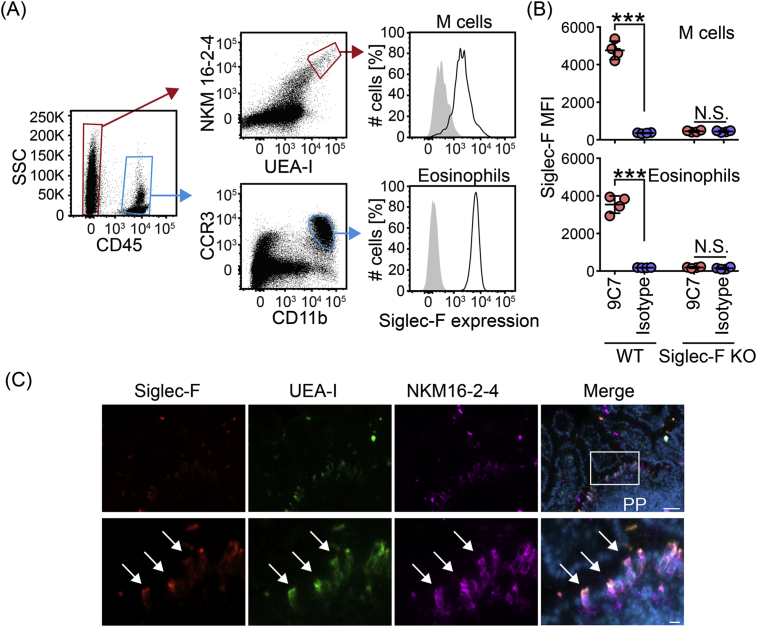
**Siglec-F expression on PP M cells**. (A) Cells isolated from the epithelial fraction of PPs were stained with anti-Siglec-F (Black) or isotype control (Grey) antibody together with the other cell surface markers. Stained cells were analyzed by flow cytometry. Dead cells were excluded from the analysis by propidium iodide staining. (B) PPs from WT and Siglec-F KO mice were analyzed for their reactivity to the Siglec-F antibody clone 9C7 as in (A). Mean fluorescent intensity (MFI) in Siglec-F staining is shown for both M cells and eosinophils. ***, p < 0.001 and N.S., not statistically significant in the Student's *t*-test (n = 4). (C) Frozen sections of PPs were stained with anti-Siglec-F antibody, UEA-I, and NKM16-2-4. Blue signals show nuclear staining with DAPI. The bottom panels are enlarged images of the luminal side of PPs (white box). White arrows show the overlapping of the three signals. White bars in the top and bottom panel are 50 μm and 10 μm, respectively. Data are representative of at least two independent experiments with similar results.

A report showing Siglec-F mRNA expression in mouse M cells [17] motivated us to investigate Siglec-F protein expression on mouse M cells using our Siglec-F monoclonal antibody (clone 9C7). PP M cells were identified as CD45^−^UEA-I^+^NKM16-2-4^+^ by flow cytometric analysis, as reported previously [17]. We found that PP M cells did indeed express Siglec-F when compared to an isotype control at levels comparable to eosinophils (CD45^+^CD11b^+^CCR3^+^) (Fig. 2A and B). Staining of M cells with the antibody clone 9C7 was specific for Siglec-F, since M cells isolated from Siglec-F KO mice stained at levels equivalent to the isotype control (Fig. 2B). In the immunohistochemical analysis, we found that Siglec-F staining was co-localized with the UEA-I and NKM16-2-4 signals in frozen sections of PP (Fig. 2C). Of note, Siglec-F expression was observed on the intestinal luminal surface of M cells (Fig 2C, arrows). Together these data demonstrate that Siglec-F is expressed on the luminal surface of mouse PP M cells.

### 3.3 *In vivo* M cell targeting by anti-Siglec-F antibody

**Fig. 3:**
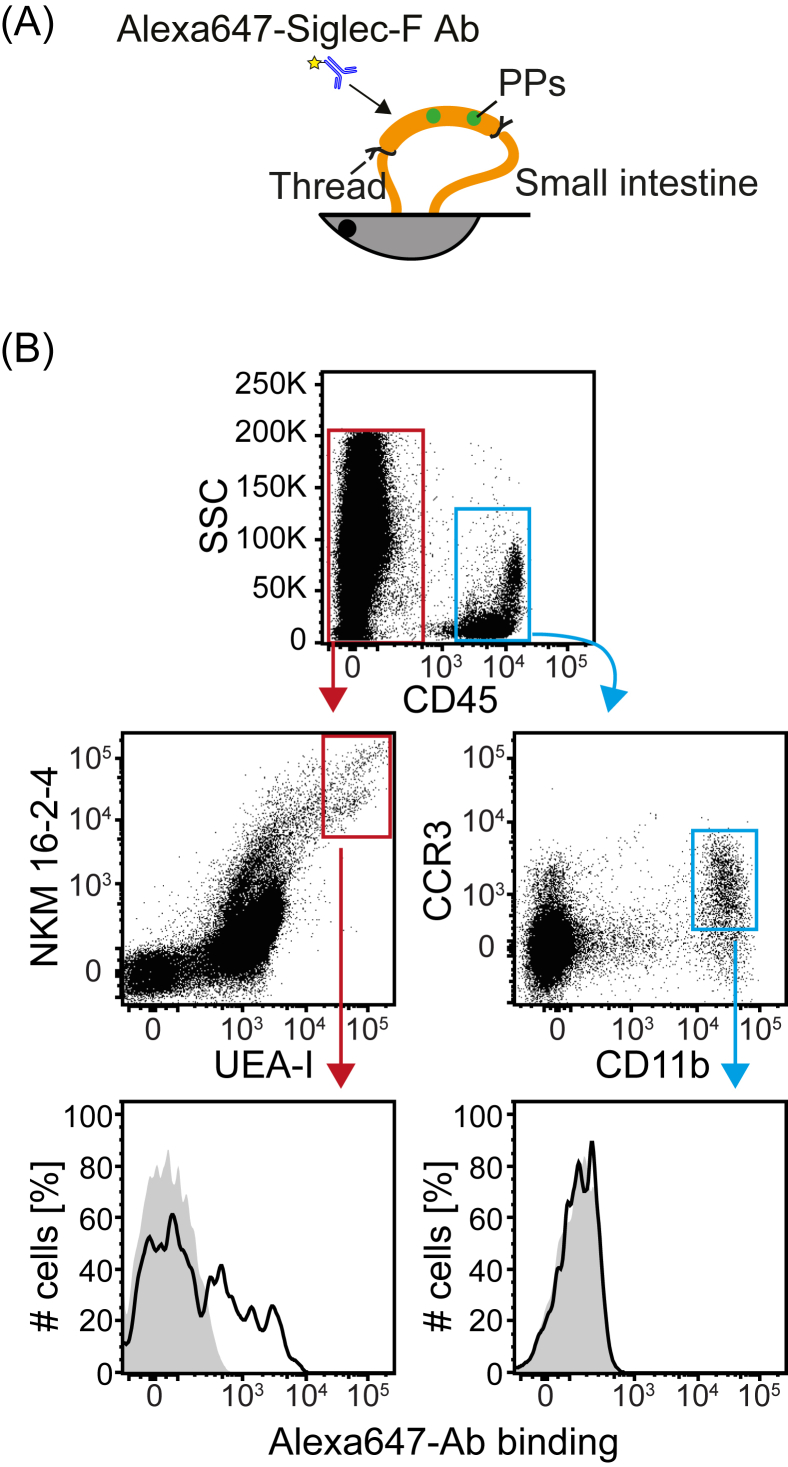
***In vivo* M cell targeting by anti-Siglec-F antibody**. Alexa647-labelled anti-Siglec-F (Black) or isotype control (Grey) antibody was injected into the small intestine of Balb/c mice. After 15 min of inoculation, PPs were harvested, and the cells were stained as in Fig. 2. Data are representative of two independent experiments with similar results.

To test whether M cells are targeted by Siglec-F antibody, Alexa647-labelled anti-Siglec-F or an isotype control antibody were injected intraluminally into a small intestine loop created by ligatures (Fig. 3A). PPs were harvested, and cells were analyzed by flow cytometry. We found that the Siglec-F antibody bound to M cells, but not to eosinophils - presumably because the eosinophils are not exposed to the intestinal lumen - demonstrating the potential of M cell targeting through Siglec-F via the intestinal lumen (Fig. 3B).

Our findings suggest a novel role for Siglec-F in M cells, opening up several key questions to be addressed; *a*) Since Siglec-F is well documented to have endocytic activity [19], to what extent might it contribute to the antigen transport by M cells? In this regard, Siglec-F has been shown to internalize the sialylated microbe *Neisseria meningitides* into the cell [19], suggesting a potential role for Siglec-F on M cells as a receptor for sialylated microorganisms in the gut. *b*) Is Siglec-F involved in survival of M cells? To determine whether the function of Siglec-F on M cells is analogous to its function in eosinophils, it will be important to test whether antibodies and glycan ligands toward Siglec-F also regulate the survival of M cells.

In humans, Siglec-8 is characterized as a functional paralogue of Siglec-F, exhibiting the same expression pattern and glycan-binding specificity [7]. Interestingly, Siglec-5 has been reported to be expressed in human M cells [11]. Therefore, it would be of interest to assess whether Siglec-5 and Siglec-F are functional paralogues on M cells.

In summary, we have identified Siglec-F expression on mouse M cells. The receptor can be targeted by antibody *in vivo*, which suggests that Siglec-F could potentially be exploited to deliver antigen to M cells. The discovery of Siglec receptors on both mouse (Siglec-F) and human (Siglec-5) M cells suggests a potentially conserved biological function for Siglecs in the gut.

## Conflict of interest statement

The authors declare that they have no conflict of interest.
